# Novel Soy Peptide CBP: Stimulation of Osteoblast Differentiation via TβRI-p38-MAPK-Depending RUNX2 Activation

**DOI:** 10.3390/nu14091940

**Published:** 2022-05-05

**Authors:** Kuaitian Wang, Xiao Kong, Mengdi Du, Wei Yu, Zhenhua Wang, Bo Xu, Jianrong Yang, Jingru Xu, Zhili Liu, Yongqiang Cheng, Jing Gan

**Affiliations:** 1 College of Life Science, Yantai University, Yantai 264000, China; wangkuaitian@163.com (K.W.); kongxiao0608@163.com (X.K.); dmd19980612@163.com (M.D.); anhuiyuw@163.com (W.Y.); skywzh@ytu.edu.cn (Z.W.); xubo168@sina.com (B.X.); edelweissyjr@163.com (J.Y.); xu19553511032@163.com (J.X.); liu12128116@163.com (Z.L.); 2 Beijing Key Laboratory of Functional Food from Plant Resources, College of Food Science and Nutritional Engineering, China Agricultural University, Beijing 100083, China

**Keywords:** bone remodeling, soy peptide CBP, osteoblast, differentiation, p38-MAPK, RUNX2

## Abstract

DEDEQIPSHPPR, the calcium-binding peptide (CBP) identified in soy yogurt, was proven to be a potential cofactor in osteoporosis prevention in our previous study, but the mechanism was unknown. In this study, the activity of alkaline phosphatase (ALP) and osteocalcin (OCN), the regulation of RUNX2, and the expression of TβRI were investigated to elucidate the underlying mechanism. The results show that CBP upregulated ALP activity and OCN concentration and increased the expression of RUNX2 and the activation of the MAPK signaling pathway. Similarly, the expression of osteogenesis-related genes in osteoblasts also increased upon CBP treatment. Moreover, the CBP-induced enhancement of ALP activity and phosphorylation levels in the p38 pathway was inhibited by treatment with a p38 inhibitor (SB203538) and TβRI inhibitor (SB431542), respectively, suggesting that p38 and TβRI were involved in the osteogenic action. Based on the signaling pathways, the intracellular calcium concentration was significantly elevated by CBP, which was correlated with the increased behavioral functions and the relative fluorescence intensity of the bone mass. These findings suggest that CBP stimulates osteoblast differentiation and bone mineralization through the activation of RUNX2 via mechanisms related to the TβRI-p38-MAPK signaling pathways, further highlighting CBP’s important potential for treating osteoporosis.

## 1. Introduction

Osteoporosis is one of the most common and prevalent diseases worldwide. It is characterized by a decrease in bone mineral density (BMD) and increase in bone fragility [1,2]. Bone formation involves a balance between bone formation by osteoblasts and bone resorption by osteoclasts. RUNX2 as a member of the Runx’s family of transcription factors, plays an important role in bone formation [3,4,5]. Several studies have proven that RUNX2 has the potential to regulate the expression of genes specific for early osteogenic differentiation, such as alkaline phosphatase (ALP) and the late differentiation-specific genes including osteocalcin (OCN) and collagen type I. In addition, several signaling pathways have been proved to be crucial for bone metabolism including the mitogen-activated protein kinase (MAPK), WNT/β-catenin and OPG/RANKL/RANK pathways [6,7,8]. Among them, the MAPK signaling pathway is an essential regulator of bone metabolism.

The majority of the compounds recently developed for osteoporosis therapy can be separated into two main categories: antiresorptive agents and bone-anabolic agents. The former is used to decrease bone resorption, thereby reducing fractures and preventing further bone loss [9,10]. However, these agents cannot repair damaged bones. The latter category, meanwhile, is the better treatment option for patients with established osteoporosis. For example, as representative antiresorptive agents, bisphosphonates were able to inhibit the enzyme farnesyl pyrophosphate synthase. Such inhibition can reduce the rate of bone resorption and decrease the risk of fractures. Meanwhile, teriparatide (PTH) [11], a representative bone-anabolic agent, has been proven to stimulate bone formation. However, it was also reported that hormone supplementation therapy was associated with an increased risk of ovarian cancer and cardiovascular diseases [12]. Therefore, there is growing interest in using dietary peptides as regulators to promote bone metabolism without side effects.

Various dietary peptides that can promote pre-osteoblasts differentiation and bone formation have been identified, including those from animal, aquatic, and plant sources. On the one hand, some peptides such as casein phosphopeptides (CPPs) [13] have been proven to regulate the proliferation and differentiation of osteoblast-like cells by controlling the activation of the calcium messenger system. However, others can combine with receptor proteins and enter cells through endocytosis to promote bone proliferation and differentiation, such as duck egg peptide (VESS), lactoferrin-derived peptide, and collagen-binding motif peptide [14,15,16].

Soymilk beverages, as functional beverages, are popular among industry beverage suppliers due to their high protein and low fat contents [17]. However, one common limitation in the development of soybean products has been the low bioavailability of calcium they contain [18]. The improvement in soymilk’s calcium bioavailability through fortification with an inorganic calcium complex was recently reported [19]. More interestingly, in our previous research, we found that fermentation could conspicuously improve the bioavailability of calcium in soybean milk. To improve the use of soymilk, a specific peptide was purified from fermented soymilk and identified as DEDEQIPSHPPR (CBP). In addition, the data demonstrated that CBP as a carrier could improve the absorption of calcium. CBP is also associated with significantly improving the ALP activity in MC3T3-E1 cells and increasing bone mass, as studied in a model of glucocorticoid-induced osteoporosis in zebrafish (GIOP), whose osteoblasts and osteoclasts function similarly to those of humans. It may play an osteogenic role by increasing the concentration of calcium in cells. However, the mechanism underlying this effect is unclear.

The CBPs possess a large number of Glu and Pro residues, similarly to duck egg white peptides (VESS), which enter cells by binding to receptor proteins on the surfaces of osteoblasts; meanwhile, fishbone peptides (KSA) [20] have been proven to promote the osteogenic differentiation of MC3T3-E1 by regulating the MAPKs signaling pathway. Therefore, we speculate that CBP enhances bone mass mainly via the upregulation of the MAPK signaling pathway after entering the cell through endocytosis.

In summary, firstly, the effect of CBP on the key osteogenesis-related genes (ALP, RUNX2, OCN, and Col-I) in MC3T3-E1 was investigated in this study. Additionally, we sought to determine the way that peptides entered the cells by adding inhibitors of the receptor proteins on the surfaces of osteoblasts. Furthermore, an ERK inhibitor, JNK inhibitor, and p38 inhibitor were added to analyze the role of the MAPK signaling pathway. Finally, the effect of CBP on bone mass in vivo was detected using a zebrafish osteoporosis model.

## 2. Materials and Methods

### 2.1. Chemicals and Reagents

Alpha modification of Eagle’s minimum essential medium (α-MEM) was purchased from Thermo Fisher Scientific (Waltham, MA, USA). Fetal bovine serum (FBS) was purchased from Corning (Corning, NY, USA). Cetylpyridinium chloride, ethyl 3-aminobenzoate methanesulfonate (MS-222), calcein, and PVDF membrances were purchased from Sigma Aldrich (Sigma, St. Louis, MO, USA). Penicillin-streptomycin, dimethyl sulfoxide (DMSO), 0.25% trypsin-EDTA solution, L-ascorbic acid, β-glycerol phosphate, prednisolone, alendronate sodium trihydrate, phosphate-buffered solution (PBS, pH 7.2–7.4, 0.01 M) and BCA Protein Assay Kit were purchased from Solarbio Life Science (Beijing, China). Additionally, 3-(4,5-cimethylthiazol-2-yl)-2,5-diphenyl tetrazolium bromide (MTT), MAPK inhibitors (SB203580, SP600125, and U0126), phenylmethanesulfonyl fluoride (PMSF, a protease inhibitor), a TβRI inhibitor (SB431542) and cell lysis buffer were purchased from the Beyotime Institute of Biotechnology (Shanghai, China). β-actin (1:1000 dilution), PKC-α (1:1000 dilution), RUNX2 (1:1000 dilution), p38 (1:1000 dilution; phosphorylation site: T180/Y182), JNK (1:1000 dilution; phosphorylation site: T183/Y185), ERK (1:1000 dilution; phosphorylation site: T202/T204), *p*-p38 (1:1000 dilution), *p*-ERK (1:1000 dilution), and *p*-JNK (1:1000 dilution) primary antibodies and a goat anti-rabbit IgG (H + L) secondary antibody (1:2000 dilution) were purchased from Cell Signaling Technology (Danvers, MA, USA).

### 2.2. Cell Culture

MC3T3-E1 cells were purchased from the National Infrastructure of Cell Line Resource (Beijing, CHN). The cells were cultured in α-MEM medium with the addition of 10% FBS and 1% penicillin-streptomycin in an incubator under 95% air and 5% CO_2_ at 37 °C. Cells were subcultured using 0.25% trypsine when the cells confluence reached 80–90%.

### 2.3. Synthesis and Verification of the Soy Peptide (CBP)

According to our latest research, the soy peptide with a high calcium affinity was identified as DEDEQIPSHPPR, which was purified from soy yogurt. In this research, the purified peptide (DEDEQIPSHPPR) was synthesized by Nan Jing Peptide Biotechnology Corporation. Ltd. (Nanjing, China) through a solid-phase procedure. The purity of the DEDEQIPSHPPR was 98% according to HPLC analysis, and the structure of the peptide was confirmed by ESI mass spectrometry.

### 2.4. Proliferation Assay of MC3T3-E1 Cell

Osteoblast proliferation of MC3T3-E1 cells was determined by the MTT method [21]. Cells were cultured on a 96-well plate at a density of 3 × 10^3^ per well, and the cells were cultured in an incubator environment with 5% CO_2_ at 37 °C for 24 h. After that, the medium was replaced, and the cells were exposed to CBP at concentrations of 0, 0.7, 7, and 70 μM for 24, 48, and 72 h. This was followed by treatment with 10 μL MTT solution (5 mg/mL in PBS) for 3 h. After the culture medium was removed, 150 μL DMSO was put into every well, and the 96-well plates were shaken for 10 min. Absorption values were measured at 570 nm (OD_570_) with a microplate reader (Molecular Devices, San Jose, CA, USA).

### 2.5. Differentiation and Mineralization Assay of MC3T3-E1

#### 2.5.1. Analysis of ALP Activity

MC3T3-E1 cells were cultured on 6-well plates at a density of 2 × 10^5^ per well and incubated in growth medium for 48 h, then the differentiation medium (50 μg/mL L-ascorbic acid and 10 mM β-glycerol phosphate in growth medium) was then changed, and the cells were incubated for 4 days, cells were pretreated with a 20 μM concentration of an inhibitor for 2 h, then the medium was changed, and the cells were supplemented with α-MEM containing the inhibitor and various concentrations of CBP and cultivated at 37 °C for 24 h. After that, the medium was removed, and the cells were rinsed with PBS buffer. The cells were lysed with 70 μL of ice-cold lysis buffer that was supplemented with PMSF per well and were broken down using a cell disruptor (BiLon 92-II, Beijing, China). The ALP activity and protein concentration of the cell lysate was measured using an Alkaline Phosphatase Assay Kit at OD_405_ and a BCA Protein Assay Kit at OD_562__._

#### 2.5.2. Analysis of OCN Activity

Cells were cultured on a 6-well plate at a density of 2 × 10^5^ per well and incubated in growth medium for 48 h. The differentiation medium was replaced, and then the cells were incubated for 11 days. Then, the cells were exposed to CBP at concentrations of 0–70 μM for 24 h. After that, the cell cultural supernatant was collected and centrifuged for 20 min (3000 rpm). The OCN concentration of the supernatant was determined with a Mouse Osteocalcin (OCN) Elisa Kit (Nanjing Jian cheng Bioengineering Institute, Nanjing, China), measuring the absorbance at OD_450_.

#### 2.5.3. Analysis of Mineralization

Cells were cultured on a 6-well plate at a density of 2 × 10^5^ per well and incubated in growth medium for 48 h. After the growth medium was removed, the cells were exposed to CBP at concentrations of 0–70 μM in the differentiation medium for 7–28 days. The differentiation medium was replaced every 3 days. After that, the cells were rinsed with PBS buffer and fixed with 70% (*v*/*v*) ethanol for 20 min. The fixed cells were stained with 1 mL of Alizarin Red (Beyotime Biotechnology, Shanghai, China) for 30 min. The cells were rinsed with H_2_O thrice and assessed with an optical microscope (Leica DMi8, Wetzlar, Germany). Then the cells were destained with 10% (*w*/*v*) cetylpyridinium chloride for 15 min. Finally, the absorbance of the destaining solution was measured with a microplate reader at 490 nm (OD_490_) [22].

### 2.6. Effect of CBP on the mRNA Expression of Osteoblastic Markers

Cells were seeded in a 6-well plate at a density of 2 × 10^5^ per well and incubated in growth medium. After 24 h, then the cells were exposed to CBP at concentrations of 0–70 μM with or without inhibitors for 6 days. After that, the total RNA was isolated using a SPARK easy Cell RNA Kit (Spark jade, Jinan, China), and the purity of the RNA preparations was assessed using the A260/280 ratio. The cDNA was created by using a SPARK script II RT Plus Kit (Spark jade, Jinan, China). RUNX2, ALP, Col-1, and OCN expression was determined by real-time qPCR using GAPDH as the endogenous control. The primers used were RUNX2 forward: 5′-AAGTGCGGTGCAAACTTTCT-3′, reverse: 5′-TCTCGGTGGCTGGTAGTGA-3′; ALP forward: 5′-AACCCAGACACAAGCATTCC-3′, reverse: 5′-GAGAGCGAAGGGTCAGTCAG-3′; Col-1 forward: 5′-AGAGCATGACCGATGGATTC-3′, reverse: 5′-CCTTCTTGAGGTTGCCAGTC-3′ and OCN forward: 5′-CCGGGAGCAGTGTGAGCTTA-3′, reverse: 5′-TAGATGCGTTTGTAGGCGGTC-3′ [23]. The fold change was analyzed as 2^−∆∆Ct^ (∆∆Ct = ∆Ct _control_ − Ct _treatment_, ∆Ct = Ct _target gene_ − Ct _Gapdh_).

### 2.7. Western Blotting Analysis

Cells were cultured on a 6-well plate at a density of 2 × 10^5^ per well for 24 h and then exposed to CBP of 0–70 μM for 0–24 h. After that, the cells were rinsed with ice-cold PBS buffer and were lysed with 70 μL ice-cold lysis buffer that was supplemented with PMSF per well. The protein was separated by 10% sodium dodecyl sulfate polyacrylamide gel electrophoresis (SDS-PAGE) and transferred to PVDF membranes [24]. The membranes were blocked with 5% skim milk (Servicebio Technology Co., Wuhan, China) and treated with the corresponding primary and secondary antibodies. The blots were identified using an enhanced chemiluminescence (ECL) kit (Beyotime Biotechnology, Shanghai, China) in conjunction with a Tanon automatic chemiluminescence imaging analysis system (Tanon, Shanghai, China). The protein band intensity quantification was determined by ImageJ software (National Institutes of Health, Bethesda, MD, USA).

### 2.8. Calcium Ion Measurement

Cells were seeded in a 6-well plate at a density of 2 × 10^5^ per well and incubated in growth medium for 24 h. Then, the cells were exposed to CBP of 0–70 μM for 48 h. The calcium concentration of the cell lysate was measured with a calcium Assay Kit (Beyotime Biotechnology, Shanghai, China) at 575 nm (OD_575_) with a microplate reader [25].

### 2.9. Assessment of Antiosteoporosis Effects in a Zebrafish Model of GIOP

In order to assess the antiosteoporosis effect of CBP, the GIOP zebrafish model was structured according to Barret [26]. Zebrafish embryos were cultured in 6-well plates. The zebrafish embryos were treated in 0.1% DMSO (vehicle), 25 μM prednisolone (model), or co-incubated 3.3–30 μM CBP, or 0.308 μM alendronate (positive control) after 3 dpf to 7 dpf. The fish water was replaced every day. Finally, calcein staining and bone mineralization of zebrafish embryos were analyzed.

#### 2.9.1. Zebrafish Husbandry and Maintenance

Adult wild-type AB-strain zebrafish were provided by China Zebrafish Resource Center (CZRC, Beijing, China). Adult zebrafish were maintained at 28.5 °C on a 14 h light/10 h dark cycle for natural mating. The zebrafish embryos and larvae were cultured at 28.5 °C in fish water (5.0 mM NaCl, 0.17 mM KCl, 0.33 mM CaCl_2_, and 0.33 mM MgSO_4_), and the suitable embryos were selected for the experiment. All experiments were performed according to the National Institutes of Health Guidelines for the Care and Use of Laboratory Animals.

#### 2.9.2. Calcein Staining and Image Acquisition

Zebrafish were exposed to fish water with 0.2% (*w*/*v*) calcein solution for 5 min. After they were washed thrice using dish water, 0.016% (*w*/*v*) MS-222 was injected, and the specimen was fixed on a depression slide using 3% (*w*/*v*) methylcellulose (Aladdin, Shanghai, China). We conducted observations with fluorescence microscopy (Leica DMi8, Wetzlar, Germany) and captured the images with digital cameras. The relative fluorescence intensity (RFI) of the skull bone mass per zebrafish was determined using the ImageJ software (*n* = 6) [27].

#### 2.9.3. Behavioral Analysis

After the CBP exposure of the larvae at 96 hpf, the spontaneous embryos were tracked for 10 min in 96-well plates at 27.5 ± 1 °C between 9 a.m. and 12 a.m., which is when the light was particularly suitable. The 7-dpf spontaneous embryo movements per minute were calculated from the tail coil alternations over a 10 min period, which was recorded using a zebrafish behavior tracking system (Danio Vision, Noldus, Wageningen, The Netherlands) [28].

### 2.10. Statistical Analysis

All the data are presented as means ± standard errors of means (SEMs) of three to six independent experiments. The data were analyzed using GraphPad Prism 5.0 (GraphPad Software, San Diego, CA, USA) and Excel (Microsoft, San Francisco, CA, USA) by one-way ANOVA with Duncan’s post-hoc test for comparisons with the vehicle. Differences with a *p*-value < 0.05 were considered to be significant.

## 3. Results

### 3.1. CBP Stimulated Cell Proliferation in MC3T3-E1 Cells

**Figure 1:**
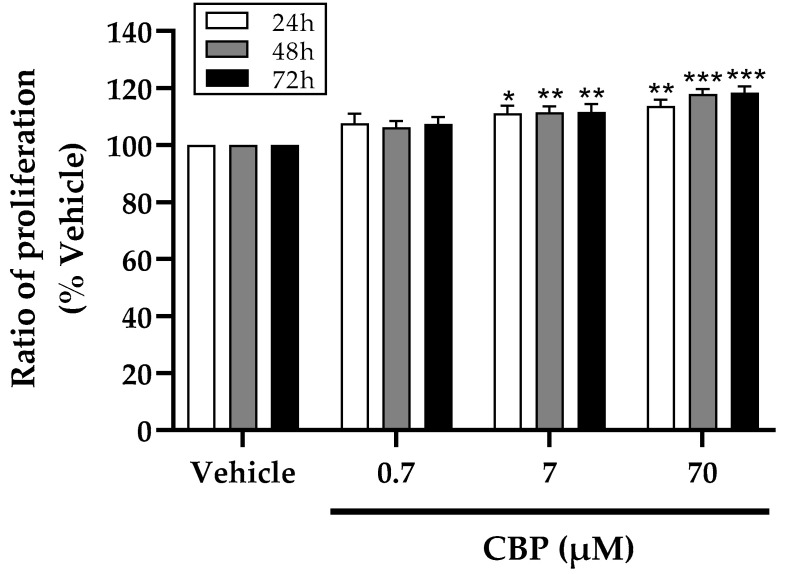
Effects of CBP on cell proliferation. CBP at concentrations of 0.7, 7, and 70 μM was tested for inducing cell proliferation at 24, 48, and 72 h. MTT assays were performed by measuring absorbance at 570 nm. *n* = 5. Data are presented as means ± SEMs and analyzed by one-way ANOVA followed by Tukey’s multiple comparison test. * *p* < 0.05, ** *p* < 0.01, and *** *p* < 0.001 vs. vehicle.

To evaluate the function of CBP in preventing osteoporosis, we firstly tested the impact of CBP on the MC3T3-E1 cells proliferation. As shown in Figure 1, various concentrations (0.7 μM, 7 μM, and 70 μM) of CBP were added to the culture media for 24 h, 48 h, and 72 h, respectively. Treatment with both 7 μM and 70 μM CBP exhibited stimulatory effects on osteoblastic cell proliferation, which were concentration-dependent manner (*p* < 0.005). Furthermore, an increase of 16.64%,17.87%, and 18.39% in cell proliferation was observed after 24, 48, and 72 h of 70 μM treatment. However, no significant stimulatory effect was observed after treatment with 0.7 μM. Meanwhile, no cytotoxic effect of CBP was observed on the osteoblast MC3T3-E1 cells was observed. These results revealed that CBP could stimulate osteoblast proliferation, showing the latent role of CBP as an osteo-inductive factor in bone formation.

### 3.2. CBP Stimulated Differentiation and Mineralization in MC3T3-E1 Cells

**Figure 2:**
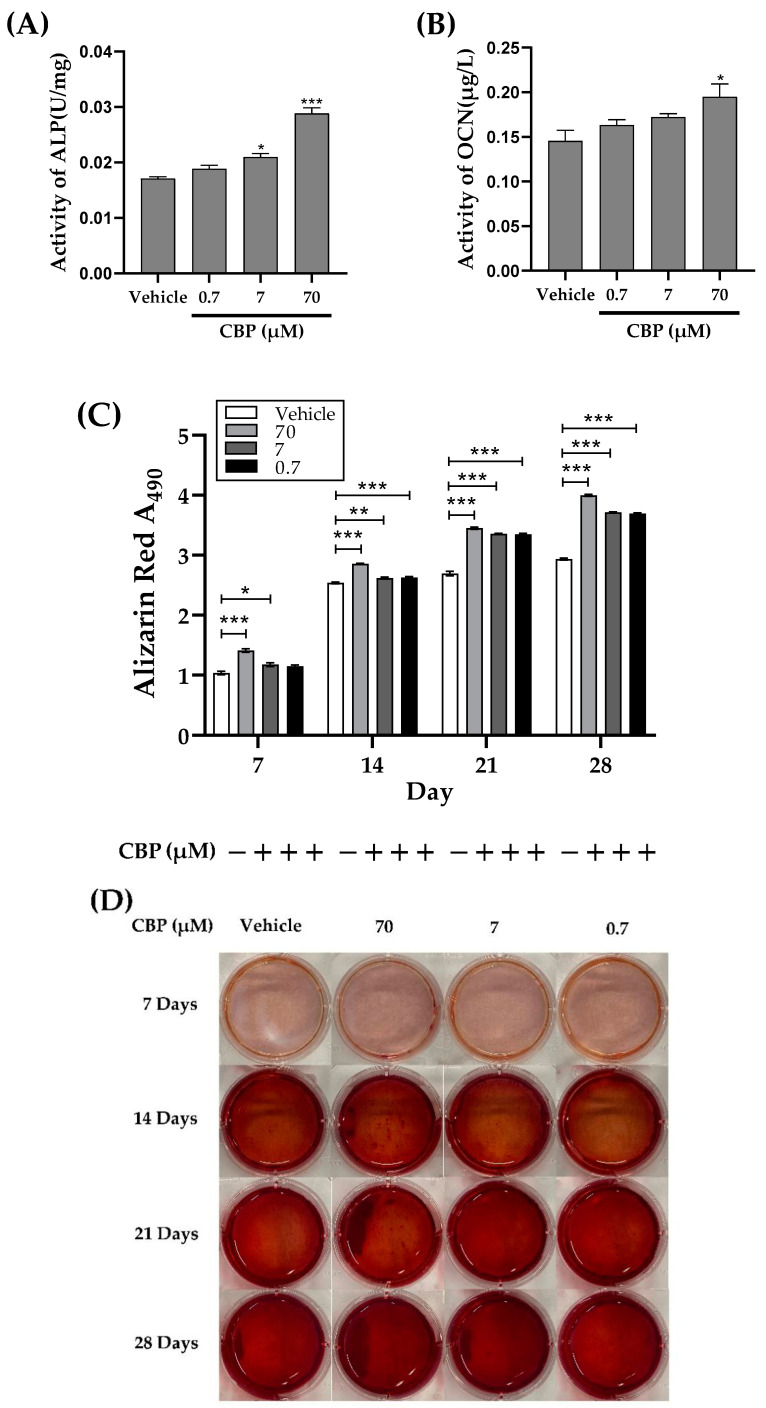
Effects of CBP on differentiation and mineralization in osteoblast cell MC3T3-E1. (**A**) Effect of CBP (0.7, 7, 70 μM) on alkaline phosphatase (ALP) activity at 24 h. (**B**) Effect of CBP (0.7, 7, 70 μM) on osteocalcin (OCN) at 24 h. (**C**,**D**) Cells were cultured with CBP (0.7, 7, 70 μM) (+) or without (−) CBP with a differentiation medium including 10 μM β-Glycerol phosphate and 50 μg/mL L-ascorbic acid for 7–28 days. Then, cells were stained with an Alizarin Red S Staining Kit, and images were captured. After cell destaining with cetylpyridinium chloride in sodium phosphate, the absorbance was measured at 490 nm. Data are mean ± SEM from 3 independent experiments. * *p* < 0.05, ** *p* < 0.01, and *** *p* < 0.001 vs. vehicle.

Cell differentiation and mineralization are the crucial steps for bone formation. The first stage of bone formation is differentiation. During the process of differentiation, the expression levels of osteoblast differentiation markers, including ALP activity and OCN concentration, directly reflect the activity and/or function of osteoblasts [28,29]. As shown in Figure 2A, the activity of ALP in MC3T3-E1 cells was increased from 0.0171 U/mg to 0.0288 U/mg after CBP treatment in a concentration-dependent manner (the range of 0.7–70 μM). Meanwhile, an increase of 33.96% in OCN activity was observed after 24 h of 70 μM CBP treatment. These results indicated that CBP possessed a potential pro-osteogenic function.

The last stage of bone formation is bone mineralization. The magnitude of the absorbance of Alizarin Red S staining is directly proportional to the amount of calcium deposition, which is a direct marker of cell mineralization. Thus, different concentrations of CBP (0.7, 7, and 70 μM) were added to MC3T3-E1 for 7, 14, 21, and 28 days, respectively. The absorbance and nodules were detected after staining with Alizarin Red S. As shown in Figure 2C,D, more obvious nodules were observed in the CBP- treatment groups in a time- and concentration-dependent manner. Generally, in the first 7 days, there was a significant difference between the untreated group and maximum dose group (70 μM), but no obvious difference among other groups, including the 0.7 and 7 μM groups. Similarly, at 14 days, a remarkable increase in absorbance was found in the 70 μM group. At 21 days and 28 days, the absorbance of the 0.7 μM, 7 μM, and 70 μM groups was significantly increased compared with that of the untreated group.

### 3.3. CBP Increased mRNA Expression of Osteoblastic Markers

**Figure 3:**
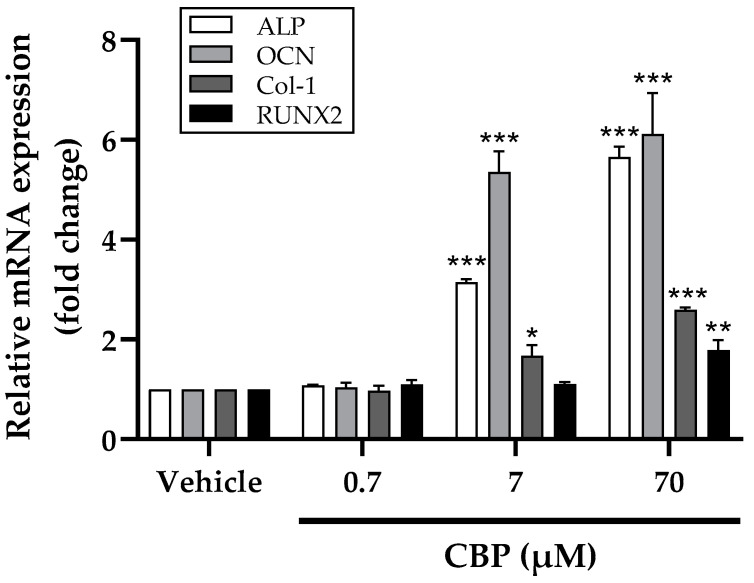
Effects of CBP on mRNA expression of bone formation markers (ALP, OCN, Col-1 and RUNX2) in MC3T3-E1 cells. MC3T3-E1 cells were treated with 0–70 µM CBP for 7 days. *n* = 3. Data are expressed as mean ± SEM. * *p* < 0.05, ** *p* < 0.01, and *** *p* < 0.001 vs. vehicle.

In order to make further explore the potential osteogenic activity of CBP, the mRNA expression of osteogenic marker genes (ALP, OCN, and Col-1) and the transcription factors RUNX2 at 7 days were examined by RT-PCR assay. The results showed that all the genes, including ALP, OCN, Col-1, and RUNX2, were upregulated in a dose-dependent manner under 7–70 µM of CBP exposure, while the effect of RUNX2 was relatively weak (Figure 3). Therefore, the RUNX2 may be the key downstream target for the promotion of osteoblast differentiation by CBP. These results revealed the key role of CBP in bone homeostasis, and the CBP at 70 µM exhibited stronger osteogenic activity; therefore, we selected 70 µM CBP treatment for further in vitro experiments.

### 3.4. CBP Promoted Osteoblast Differentiation by Activating the MAPK Pathway

**Figure 4:**
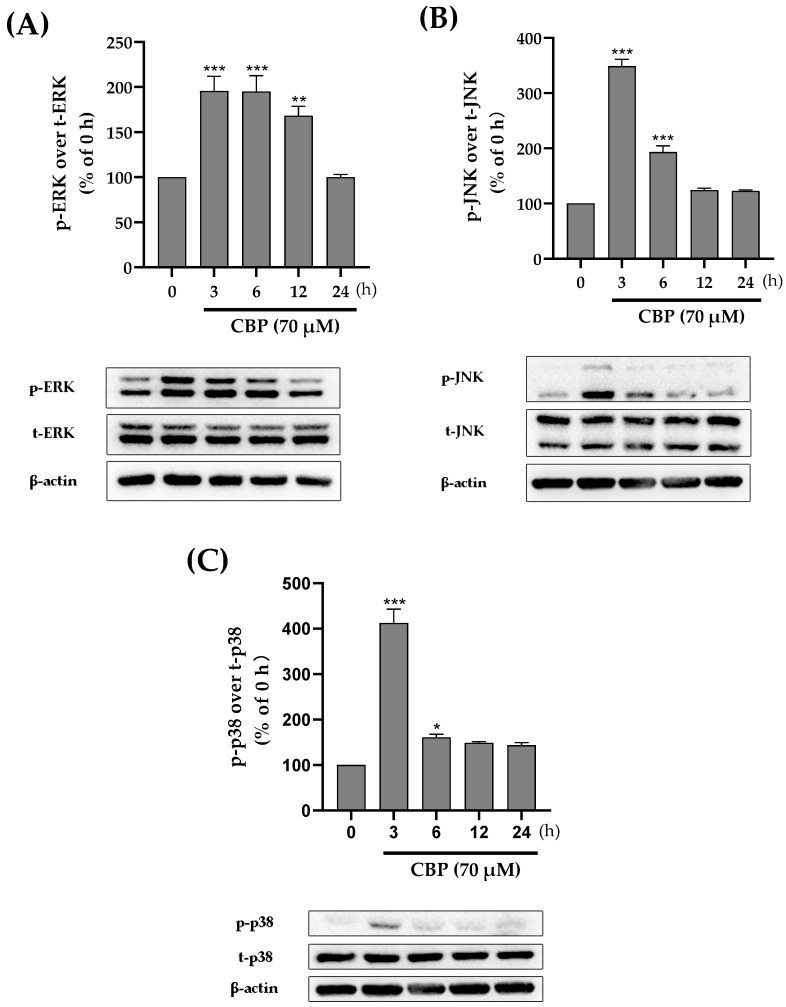
Effects of CBP on phosphorylation levels of ERK, JNK, and *p*-38 signaling pathways expression. (**A**) Phosphorylation level of ERK signaling pathway in MC3T3-E1 cells cultured to 70 μM CBP for 0–24 h. (**B**) Phosphorylation level of the JNK signaling pathway in MC3T3-E1 cells cultured to 70 μM CBP for 0–24 h. (**C**) Phosphorylation level of p38 signaling pathway in MC3T3-E1 cells cultured to 70 μM CBP for 0–24 h. *n* = 3. Data are expressed as mean ± SEM. * *p* < 0.05, ** *p* < 0.01, and *** *p* < 0.001 vs. vehicle.

A number of factors influence the regulation of osteoblast proliferation and osteogenic differentiation. For example, the MAPK family, including ERK, JNK, and p38, has been proven to regulate the differentiation of osteoblasts [30,31]. The MAPK pathway has also been shown to be related to bone-mass modulation. Therefore, to explore the role of ERK, JNK, and p38 in CBP-induced osteoblast differentiation, we investigated the phosphorylation status of ERK, JNK, and p38. The results showed that 70 μM CBP dramatically increased ERK, JNK, and p38 phosphorylation within the initial 3 h, respectively. After this rapid activation, the phosphorylation of JNK and p38 gradually declined to the basal level, but the ERK decreased to the basal level after 6 h (Figure 4A–C). Interestingly, once pathways including ERK, JNK, and p38 were activated by CBP, the phospho-ERK, phospho-JNK, and phospho-p38 were translocated into the nuclei. All the results showed that CBP dramatically activated the ERK, JNK, and p38.

### 3.5. CBP Stimulated Osteoblastic Differentiation Activity through the TβRI-p38-MAPK-Mediated Activation of RUNX2 Pathway

**Figure 5:**
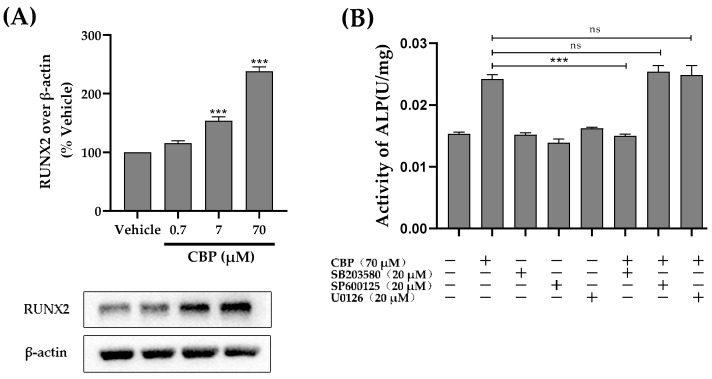

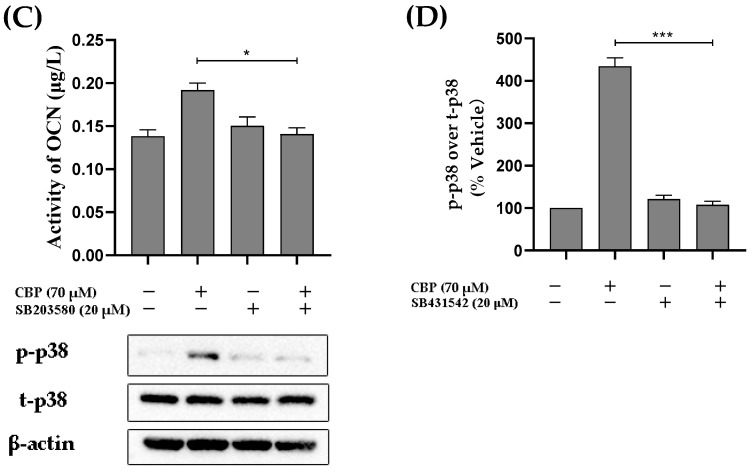
CBP promotes osteoblastic differentiation through the p38-MAPK mediation of the RUNX2 pathway. (**A**) CBP treatment (0.7, 7, 70 μM) for 24 h raises the expression of RUNX2. (**B**) Effect of MAPK inhibitor (SB203580, SP600125, and U0126) (+) and culture medium (−) on CBP treatment (70 μM) for 24 h increases ALP activity in MC3T3-E1. (**C**) Effect of p38 pathway inhibitor (SB203580) (+) and culture medium (−) on CBP treatment (70 μM) for 24 h raises OCN concentration in MC3T3-E1. (**D**) Phosphorylation level of p38 signaling pathway in MC3T3-E1 cells cultured to 70 μM CBP and with (+)TβRI inhibitor (SB431542) or without (−)TβRI inhibitor (SB431542) for 3 h. *n* = 3. Data are expressed as mean ± SE. * *p* < 0.05, *** *p* < 0.001, ns, not significant.

Runt-related transcription factor 2 (RUNX2), as a multifunctional transcription factor, plays an important roles in osteoblast differentiation [32,33]. We investigated the protein expression of the transcription factor RUNX2 downstream of the MAPK pathway (Figure 5A). The results reveal that CBP could activate the ERK, JNK, or p38 signaling pathway. However, it was unclear which signaling pathways were associated with the osteogenic effects of CBP. Therefore, a pharmacological inhibition test was used to further explore the role of CBP in accelerating the osteogenic differentiation of MC3T3-E1 cells. A U0126 inhibitor, SP600125 inhibitor, and SB203580 inhibitor were used to prevent the individual activation of the ERK, JNK, and p38-MAPK pathways [34]. The results showed that MC3T3 cells treated with 20 μM SB203580 for 24 h exhibited a dramatic decrease in the activity of ALP, which was comparable to that observed with CBP treatment alone (Figure 5B). However, treatment with the U0126 inhibitor and SP600125 inhibitor did not affect the activity of ALP in MC3T3 cells. Moreover, the addition of the p38 inhibitor (SB203580) significantly decreased the concentration of OCN, which was increased by CBP (Figure 5C). Collectively, these results suggest that CBP activates osteoblast differentiation via the p38-MAPK-RUNX2 axis and regulates its downstream effectors.

It is known that the p38 pathway is a noncanonical TGF-β pathway [35]. Previous studies have found that peptides could promote osteogenesis through binding to the TGF-β receptor, such as lactoferrin, which binds to TGF-β receptor II, promoting osteogenesis. In this work, we investigated the role of TGF-β in CBP-mediated p38-MAPK activation. In this set of experiments, the cells were treated with CBP in the presence or absence of the TβRI-specific inhibitor SB431542. The results showed that the TβRI inhibitor SB431542 was able to inhibit the CBP-mediated activation of p38-MAPK phosphorylation (Figure 5D). These results indicated that TGF-β signaling participated in the effect of CBP-induced proliferation and differentiation in osteoblast cells.

### 3.6. CBP Activated the PKCα and Ca^2+^ Pathway

**Figure 6:**
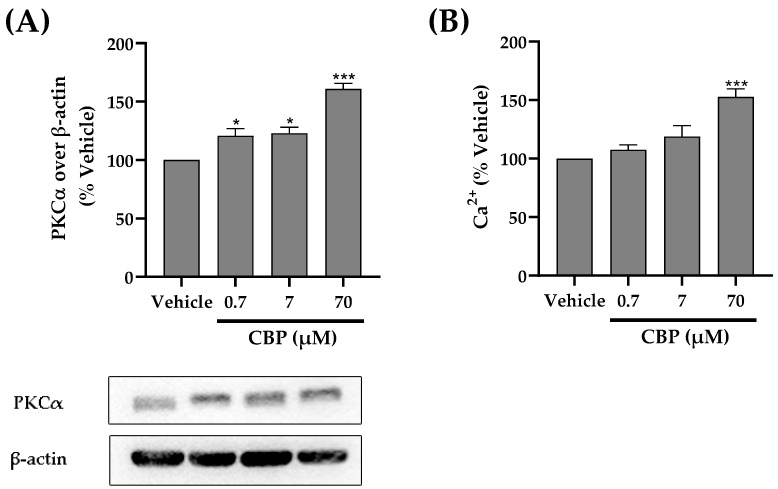
Effect of CBP on osteogenesis markers (**A**) PKCα and (**B**) Ca2^+^ signaling in MC3T3-E1 cells. MC3T3-E1 cells were cultured with CBP (0.7–70 μM) for 24 or 48 h. Western blot (PKCα) and ELISA (Calcium Assay Kit) were used to assess the expression of selected biomarkers. *n* = 3. Data are expressed as mean ± SEM. * *p* < 0.05, *** *p* < 0.001 vs. vehicle.

Calcium signaling, one of the osteogenic pathways, is regulated by the protein kinase Cα (PKCα) [36]. This peptide contains many acidic amino acids, which can bind more calcium; thus, we hypothesize that PKCα regulates CBP’s involvement in osteoblast function. The results showed that CBP remarkably increased the expression of PKCα in a dose-dependent manner, showing a 1.7-fold increase for 70 μM CBP treatment in MC3T3 cells compared to the vehicle groups. Similarly, an increased Ca^2+^ concentration after CBP treatment (70 μM) was observed (Figure 6A,B).

### 3.7. The Prevention of Osteoporosis In Vivo

**Figure A1:**
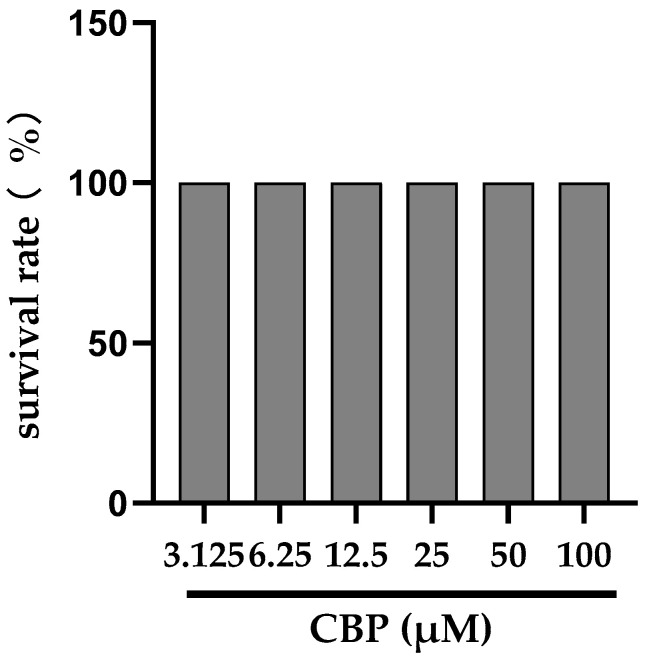
Viability of zebrafish treated with CBP.

To investigate the effect of CBP on the bone regeneration process in vivo, the toxicity of CBP was first investigated. The CBP was not lethal to zebrafish with a concentration up to the maximum solubility (0–100 μM). Therefore, concentrations up to 30 μM were safe for determining the effect of CBP on the prednisolone-treated larval zebrafish model (Figure A1).

**Figure 7:**
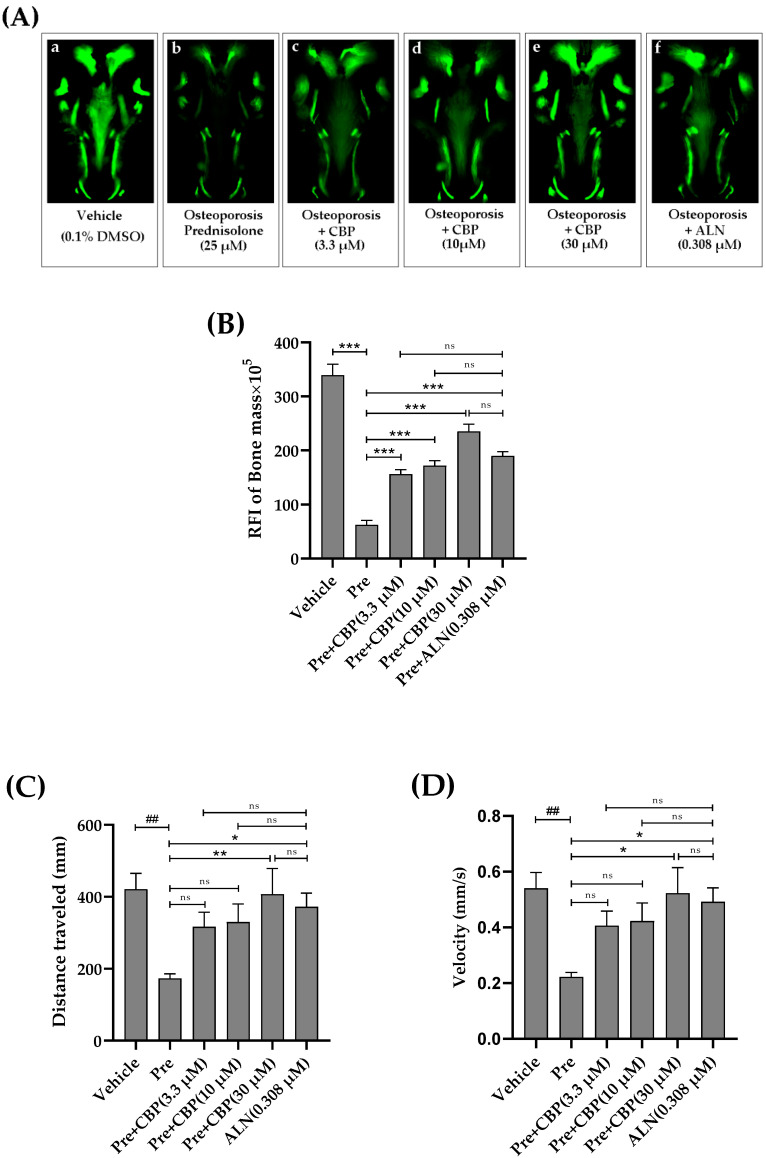
Effects of CBP on antiosteoporosis in the zebrafish model of GIOP. (**A**) Quantification of the relative fluorescence intensity (RFI) of the zebrafish skull at 7 dpf. (**B**) Representative fluorescence images of the zebrafish skull with different samples at 7 dpf (**a**) 0.1% DMSO (Vehicle), (**b**) 25 μM prednisolone (Osteoporosis), or co-administered with CBP (3.3 μM, 10 μM, 30 μM) (**c**–**e**), or 0.308 μM alendronate (positive control) (**f**) for 96 h. (**C**) Distance traveled of zebrafish larvae at 96 hpf after exposure to CBP. (**D**) Swimming speed of zebrafish larvae at 96 hpf after exposure to CBP. *n* = 10. Data are expressed as mean ± SEM. ## *p* < 0.01 vs. vehicle, * *p* < 0.05, ** *p* < 0.01, and *** *p* < 0.001 vs. Pre, ns, not significant. (Pre, prednisolone; ALN, alendronate).

Alendronate (ALN) was used as a positive control drug, and CBP (3.3, 10, and 30 μM) was added. As shown in Figure 7A, compared with the normal control group, the fluorescence intensity of the zebrafish skull in the model group was significantly reduced. Compared with the model control group, 0.308 μM of ALN reversed the decrease in fluorescence intensity. The data indicated that the GIOP model was successful.

Then the zebrafish were treated with different concentrations of CBP and vehicle solvent during the fracture repair period. The results showed that the amount of stained mineralized tissue of larvae with 25 μM prednisolone was significantly lower than that for the vehicle group (0.1% DMSO) after 3 dpf to 7 dpf (Figure 7A(a,b)). Similarly, both treatments with different concentrations of CBP (3.3,10, and 30 μM) and 0.308 μM of ALN markedly decreased the bone loss. The RFI of the bone mass of the prednisolone group also significantly decreased more than that of the vehicle group (*p* < 0.001). Meanwhile, we found that CBP significantly increased the RFI of the bone mass in a dose-dependent manner (*p* < 0.001) (Figure 7B).

As shown in Figure 7C,D, when compared with the normal control group, prednisolone elicited an obvious reduction in the distances traveled and average swimming velocity. Compared with the model control group, 0.308 μM of ALN reversed the increase in distances traveled and swimming velocity. When CBP (30 µM) was added to zebrafish larvae, the total distances were significantly increased from 173.33 to 407.32 mm. Moreover, with an increase in the CBP concentration, the average swimming velocity of the zebrafish gradually increased from 0.222 to 0.523 mm/s.

## 4. Discussion

Osteoporosis, as the most common bone disease worldwide, is usually diagnosed after a fracture occurs. Disruption of bone remodeling, including bone formation cell osteoblasts and bone resorption cell osteoclast, contributed to the occurrence of osteoporosis [37]. Recently, various kinds of efforts have been made to help with anti-resorption and bone formation [38]. CBP has been recognized as a potential activity factor that promotes bone formation. In our previous study, we purified one soybean peptide CBP (DEDEQIPSHPPR) from soy yogurt that had the ability to inhibit the degradation by protease in vivo and significantly promoted osteoblast ALP activity. However, the underlying mechanism is unclear. Herein, we investigated the roles of this identified peptide in the inhibition of osteoporosis. Different doses (0.7 μM, 7 μM, and 70 μM) of CBP have been used to investigate the proliferation, differentiation, and mineralization of MC3T3-E1 cells. Moreover, the zebrafish model of GIOP has been used to figure out the effect of CBP on bone density. To the best of our knowledge, this is the first study of that CBP’s stimulation of osteoblast differentiation and bone mineralization through the activation of RUNX2 via the TβRI-p38-MAPK signaling pathways. Meanwhile, we found that CBP can carry many calcium ions into cells through calcium channels to promote osteoblast differentiation as well.

The proliferation and differentiation of osteoblasts play an important role in bone formation and metabolism [39]. Various bone-derived proteins and bone markers, including ALP and OCN, have been shown to relate to the differentiation of osteoblasts. First, to figure out the effect of CBP on cell proliferation, our isolated CBP has been reported to promote the proliferation of osteoblasts in a dose-dependent manner. Second, ALP activity and OCN concentration of osteoblasts were upregulated significantly upon CBP treatment (70 μM) (*p* < 0.05). Meanwhile, the mineralization of the matrix was qualitatively determined by the alizarin reds staining kit, results of which suggested 70 μM CBP can help improve the mineralization significantly (*p* < 0.05) after 7 days and 14 days of incubation. All these results suggested that CBP is an effective factor for osteoblast differentiation and mineralization.

In addition, RUNX2 is the key transcription factor, and its expression level can affect osteoblast differentiation and mineralization as well. To uncover the specific mechanism of CBP in osteoblast differentiation and mineralization, mRNA expression of various osteogenesis-related genes, including ALP, OCN, and CoI-1, and the transcription factors RUNX2 after treatment with CBP have been studied. Generally, all the mRNA expression levels have been shown to be upregulated and induced by 70 μM CBP. Interestingly, this activation exhibited a dose-dependent nature for RUNX2 during treatment with 0.7–70 µM, indicating that CBP induced osteogenic differentiation through RUNX2. This is consistent with previous reports that peptides induced osteoblast differentiation via RUNX2 [34].

RUNX2 expression has been reported to relate to MAPK pathways, including ERK, JNK, and p38 pathways [40]. Thus, we propose that our isolated CBP may stimulate RUNX2 via the MAPK pathway and then promote the differentiation and mineralization of the matrix. To verify this hypothesis, the effect of CBP on the expression of tight junction proteins in the ERK, JNK, and p38 pathways was detected by Western blotting. The results show that the phosphorylation level of ERK, JNK, and p38 was significantly increased within the initial 3 h after CBP treatment (*p* < 0.05), indicating that CBP induced osteogenic differentiation via RUNX2-MAPK. However, it is thus intriguing which pathway (ERK, JNK, or p38) acts as a link between the cell surface and nucleus to regulate cell differentiation. To figure it out, the influence of CBP on ALP activity in MC3T3 cells was determined when adding inhibitors of ERK, JNK, and p38 individually. Results showed that blocking ERK with a U1026 inhibitor and blocking JNK with SP600125 did not affect the CBP-induced enhancement of ALP activity in osteoblasts. However, blocking p38 with an SB203580 inhibitor significantly decreased the CBP-induced stimulation of ALP activity in osteoblasts (*p* < 0.05). Therefore, the p38 pathway is recognized as one of the most fundamental mechanisms by which CBP improves the activation of RUNX2, thus regulating the differentiation process. It is well known that the p38 pathway is a noncanonical TGF-β pathway [35]. Lactoferrin has been proved to promote osteogenesis through binding to the TGF-β receptor. Additionally, previous studies have suggested that peptides, such as YRGDVVPK purified from *Crassostrea gigas*, have been proven to promote pre-osteoblast proliferation through to surface receptor proteins on MC3T3-E1 [41]. To verify whether CBP enters the cell through binding with TGF-β receptor II on the cell surface, the phosphorylation levels of p38 have been determined. The results showed that phosphorylation of p38 has been inhibited by TβRI inhibitor SB431542, suggesting that the downstream signal transduction in these cells is primarily mediated by TβRI.

In addition, several peptides, such as CPP, have the ability to transfer more calcium into cells and promote the formation of new bones [13]. CBP was identified as a high calcium-chelating peptide in our previous study. In the present study, we showed that the concentration of intracellular calcium ions was greatly increased by CBP, suggesting that activation of calcium ions transport in cells may be another reason for the induction of osteoblast differentiation.

Lastly, to better understand the relationship between CBP and bone formation, the zebrafish GIOP model was used to evaluate CBP’s effects on osteoporosis. The results confirmed that 70 μM CBP is able to promote the formation of new bone significantly (*p* < 0.05), which is much better than previously reported antimicrobial peptides from a marine fish [42].

## 5. Conclusions

To summarize, this is the first study indicating that naturally derived CBP stimulates the osteoblast differentiation of MC3T3 cells mainly through the TβRI-p38-MAPK-RUNX2 signaling pathway, but not the ERK or JNK pathway. An increase in the intracellular concentration of free calcium is another factor by which CBP exhibits osteogenic activity. This finding contributes to a new understanding with regard to the molecular mechanisms underlying the activating effect of CBP on bone and will promote the further use of CBP as a natural antiosteoporosis reagent.

## Data Availability

Not applicable.

## Abbreviations

CBP	soy peptide
DEDEQIPSHPPR	Asp-Glu-Asp-Glu-Gln-IIe-Pro-Ser-His-Pro-Pro-Arg
ALP	Alkaline phosphatase
